# HIV service delivery models towards ‘Zero AIDS-related Deaths’: a collaborative case study of 6 Asia and Pacific countries

**DOI:** 10.1186/s12913-015-0804-5

**Published:** 2015-04-24

**Authors:** Masami Fujita, Krishna C Poudel, Kimberly Green, Teodora Wi, Iyanthi Abeyewickreme, Massimo Ghidinelli, Masaya Kato, Mean Chhi Vun, Seng Sopheap, Khin Ohnmar San, Phavady Bollen, Krishna Kumar Rai, Atul Dahal, Durga Bhandari, Peniel Boas, Jessica Yaipupu, Petchsri Sirinirund, Pairoj Saonuam, Bui Duc Duong, Do Thi Nhan, Nguyen Thi Minh Thu, Masamine Jimba

**Affiliations:** World Health Organization Cambodia, P.O. Box 1217, , No. 177-179 Pasteur (St.51), Sangkat Chak Tomouk, Phnom Penh Cambodia; Department of Health Promotion and Policy, School of Public Health and Health Sciences, University of Massachusetts Amherst, Amherst, USA; Formerly FHI 360, Accra, Ghana; World Health Organization, Geneva, Switzerland; Formerly World Health Organization Regional Office for South-East Asia, New Delhi, India; Pan American Health Organization, World Health Organization Regional Office for the Americas, Washington, DC USA; World Health Organization, Hanoi, Vietnam; National Center for HIV/AIDS, Dermatology and STD, Ministry of Health, Phnom Penh, Cambodia; Formerly National AIDS Program, Ministry of Health, Nay Pyi Taw, Myanmar; World Health Organization, Yangon, Myanmar; National Center for AIDS and STD Control, Ministry of Health, Kathmandu, Nepal; World Health Organization, Kathmandu, Nepal; FHI 360, Kathmandu, Nepal; STI, HIV and AIDS Surveillance Unit, Ministry of Health, Port Moresby, Papua New Guinea; World Health Organization, Port Moresby, Papua New Guinea; National AIDS Management Center, Ministry of Public Health, Bangkok, Thailand; Vietnam Authority of HIV/AIDS Control, Ministry of Health, Hanoi, Vietnam; Clinton Health Access Initiative, Hanoi, Vietnam; Department of Community and Global Health, Graduate School of Medicine, University of Tokyo, Tokyo, Japan

**Keywords:** HIV, Service delivery, Care continuum, Case detection, Retention in care, HIV cascade, Asia-Pacific

## Abstract

**Background:**

In the Asia-Pacific region, limited systematic assessment has been conducted on HIV service delivery models. Applying an analytical framework of the continuum of prevention and care, this study aimed to assess HIV service deliveries in six Asia and Pacific countries from the perspective of service availability, linking approaches and performance monitoring for maximizing HIV case detection and retention.

**Methods:**

Each country formed a review team that provided published and unpublished information from the national HIV program. Four types of continuum were examined: (i) service linkages between key population outreach and HIV diagnosis (vertical-community continuum); (ii) chronic care provision across HIV diagnosis and treatment (chronological continuum); (iii) linkages between HIV and other health services (horizontal continuum); and (iv) comprehensive care sites coordinating care provision (hub and heart of continuum).

**Results:**

Regarding the vertical-community continuum, all districts had voluntary counselling and testing (VCT) in all countries except for Myanmar and Vietnam. In these two countries, limited VCT availability was a constraint for referring key populations reached. All countries monitored HIV testing coverage among key populations.

Concerning the chronological continuum, the proportion of districts/townships having antiretroviral treatment (ART) was less than 70% except in Thailand, posing a barrier for accessing pre-ART/ART care. Mechanisms for providing chronic care and monitoring retention were less developed for VCT/pre-ART process compared to ART process in all countries.

On the horizontal continuum, the availability of HIV testing for tuberculosis patients and pregnant women was limited and there were sub-optimal linkages between tuberculosis, antenatal care and HIV services except for Cambodia and Thailand. These two countries indicated higher HIV testing coverage than other countries.

Regarding hub and heart of continuum, all countries had comprehensive care sites with different degrees of community involvement.

**Conclusions:**

The analytical framework was useful to identify similarities and considerable variations in service availability and linking approaches across the countries. The study findings would help each country critically adapt and adopt global recommendations on HIV service decentralization, linkages and integration. Especially, the findings would inform cross-fertilization among the countries and national HIV program reviews to determine county-specific measures for maximizing HIV case detection and retention.

## Background

The HIV epidemic is at a critical juncture in Asia and the Pacific region. In this region, remarkable progress has been made as indicated by a 26% decline in new HIV infections from 2001 to 2012, and a 46% increase in access to antiretroviral therapy (ART) from 2009 to 2012, reaching overall HIV treatment coverage of 51% [1]. However, the majority of the low and middle-income countries in the region need more time to achieve the global goals described in Zero New HIV Infections, Zero AIDS-related Deaths, and Zero Discrimination [2-4].

To reach Zero AIDS-related Deaths, the Treatment 2.0 initiative gives us hope. It aims to stimulate innovation and improve the efficiency and impact of HIV prevention, care and treatment programs in resource-limited countries [5,6]. One of the five pillars of Treatment 2.0 is to “adapt delivery systems”. This pillar calls for decentralization and for integrating HIV care and treatment with other HIV and non-HIV services such as drug dependency services, maternal, newborn and child health (MNCH), or tuberculosis (TB) services. The primary aims of this pillar are; (a) increasing community engagement for HIV testing and counseling, (b) promoting early enrollment in pre-ART care and ART, and (c) retaining them for life. This initiative calls for the expanding evidence base on optimal service delivery models in a variety of settings and in resource-limited contexts.

To date, systematic assessment has been rarely conducted on overall HIV service delivery models and HIV service delivery analyses have been fragmented. Its focus has been on either one component of HIV health services such as ART [7], or integration between two services such as HIV and TB [8], HIV and MNCH [9] or HIV and family planning [10].

In Asia and the Pacific region, the HIV epidemic is concentrated among key populations such as female sex workers (FSWs), men who have sex with men (MSM), transgender (TG) and people who inject drugs (PWID). The continuum of care concept has been used by HIV programs to coordinate and link health facilities, the community, and other sectors under one coherent framework [11-15]. The continuum of care has been more recently understood as the continuum of prevention and care (COPC). The COPC can be regarded as a coordinated network of prevention, treatment, care, and support activities across different levels of the health system including the community, resulting in provision of comprehensive services over the long-term [15]. The COPC contributes to prevention of HIV infections, HIV case detection and retention in care across the HIV cascade [16,17].

An analytical framework based on the COPC concept was recently proposed and applied to assess HIV service delivery in Vietnam [18]. The assessment focused on analyzing availability of HIV related services including geographical distribution and decentralization, and approaches to link between the target populations and the services and among the services across four types of continuum. These continuum were: (i) local coordination mechanisms and comprehensive care site (Hub and Heart of Continuum); (ii) chronic care provision throughout the stages of HIV diagnosis, pre-ART care, ART and end-of-life care (Chronological Continuum); (iii) linkages and/or integration across HIV and other health services and across different geographical administrative areas (Horizontal Continuum); and (iv) service linkages across community and different levels of health facilities (Vertical-Community Continuum).

The assessment identified system-related strengths and constraints of the country’s HIV service delivery for improving HIV case detection and retention in care. Strengths included decentralized HIV service delivery with good linkages in high burden provinces. Constraints included centralized HIV service delivery with limited linkages in middle/low burden provinces as well as lack of mechanism to monitor the linkages.

We assumed that applying the COPC analytical framework to multiple countries with concentrated HIV epidemics could characterize the HIV service delivery models for cross-fertilization and optimization. This study aimed to assess HIV service deliveries in six Asia and Pacific countries from the perspective of service availability, linking approaches and performance monitoring with a view to maximizing HIV case detection and retention.

## Methods

### Background information of the study countries

The study countries are Cambodia, Myanmar, Nepal, Papua New Guinea (PNG), Thailand and Vietnam. These countries were chosen based on the population size (5 to 100 millions), area (100,000 to 1,000,000 square kilometer), income level (low-income to upper-middle), type of HIV epidemics (concentrated), and willingness of the national HIV programs, World Health Organization (WHO) country offices, and FHI360 country offices to participate in the study.

Among these countries, Thailand was categorized as an upper middle-income country and PNG and Vietnam were lower middle-income countries while the remaining three were categorized as low-income countries (see Table 1 for the characteristics of study countries, including population, HIV prevalence, and service coverage). By 2010, Cambodia and Thailand had reached high ART coverage; PNG and Vietnam just surpassed 50%; while Nepal and Myanmar remained far below 50% [19-24]. Myanmar and Thailand had achieved higher prevention of mother-to-child transmission (PMTCT) coverage than ART coverage.

**Table 1:** **Brief characteristics of the study countries**

	**Cambodia**	**Myanmar**	**Nepal**	**PNG**	**Thailand**	**Vietnam**
**General information**						
Total area (sq km)	181,035	678,500	136,801	459,854	514,000	329,560
Population (m.) (2009)	15	50	29	7	68	87
No. of provinces/Zones	24	14	14	20	76	63
No. of districts/Townships	77	330	75	89	878	697
Adult literacy rate (%) (2008)	78	92	58	60	94	93
Income level	Low	Low	Low	Lower middle	Upper middle	Lower middle
GNI per capita (US$) (2009)	650	-	440	1,180	3,760	1,010
**HIV epidemic**						
Estimated number of PLHIV as of 2010	56,200	226,000	70,000	54,000	517,000	254,000
HIV prevalence**						
Adults (aged 15–49)	0.6 (2011)	0.53 (2011)	0.3 (2011)	0.8 (2011)	1.3	0.4
People who inject drugs (PWID)	24.4 (2007)	21.9 (2011)	6.3 (2011)	Not available	38.7 (2009)	18.4 (2009)
Female sex workers (FSW)	13.9 (2010)	9.4 (2011)	1.7 (2011)	17.8 (2010)	2.8 (2009)	3.2 (2009)
Men who have sex with men (MSM)	2.1 (2010)	7.8 (2011)	3.8 (2009)	Not available	13.5 (2009)	16.7 (2009)
HIV program coverage**						
ART coverage (%)	89.5 (2011)	43.8 (2011)	23.7 (2011)	61.2 (2011)	64.6 (2011)	54.0 (2011)
HIV-positive women who received ARV to reduce the risk of mother-to-child HIV transmission (%)	63.5 (2011)	54.5 (2010)	12.2 (2011)	12.3 (2011)	94.0 (2011)	44.0 (2011)
TB cases who received ART and TB treatment (%)	32.7 (2011)	Not available	Not available	25.1 (2011)	29.8 (2011)	30.1 (2011)
Key populations reached by HIV prevention program**						
Female sex workers (%)	81.5 (2010)	76.2 (2008)	60.0 (2011)	36.4 (2010)	56.9 (2010)	47.3 (2009)
Men who have sex with men(%)	69.5 (2010)	69.1 (2009)	77.3 (2009)	66.6 (2010)	49.2 (2010)	24.0 (2009)
People who inject drugs (PWID) (Number syringe distributed per injecting drug user per year)	120.2 (2011)	123 (2011)	71.4 (2011)	Not available	9.8 (2010)	140 (2011)
HIV testing coverage among key populations**						
Female sex workers	81.5 (2010)	71.1 (2008)	54.6 (2011)	46.4 (2010)	50.4 (2010)	43.8 (2011)
Men who have sex with men	34.0 (2010)	47.6 (2009)	42.0 (2009)	55.8 (2010)	29.2 (2010)	30.2 (2011)
People who inject drugs	35.3 (2007)	27.3 (2008)	21.4 (2011)	Not available	40.8 (2010)	29.1 (2011)

Remark: GNI: Gross national income.

**AIDS Progress Report, 2012 of Cambodia [19], Myanmar [20], Nepal [21], PNG [22], Thailand [23], and Vietnam [24].

### Analytical framework

The COPC originates from the concept of the continuum of care developed in the 1970s to offer continuity of care for the elderly [25]. The continuum of care was then applied to individual case management of various health problems for strengthening service linkages and minimizing lost to follow-up. This concept was also applied for maternal, neonatal and child health [26]. In the 1990s, the continuum of care was introduced to HIV care [27,28]. Then in 2000s, the continuum of care was used to develop a common framework to coordinate multiple stakeholders in improving access to, and retention of ART for its scaling-up. In particular, the continuum of care facilitated linkages between health facility-based services and community- and home-based care (CHBC) and promoted the involvement of people living with HIV (PLHIV) [12-15]. The concept has further evolved to strengthen the linkages and integration between HIV prevention, care and treatment [29,30].

A critical element of the COPC is to establish a comprehensive care site as a central mechanism of a local service network. The comprehensive care site offers not only clinical care but a wide range of associated services. Such services include health education, psychosocial support, links to other services and CHBC, as well as opportunities for the involvement of affected communities such as key populations and PLHIV. The names given to the comprehensive care site differ across Asia, such as the Day Care Centre, the Comprehensive Continuum of Care Centre, and the Friend-Help-Friend Centre [13,15].

**Figure 1:**
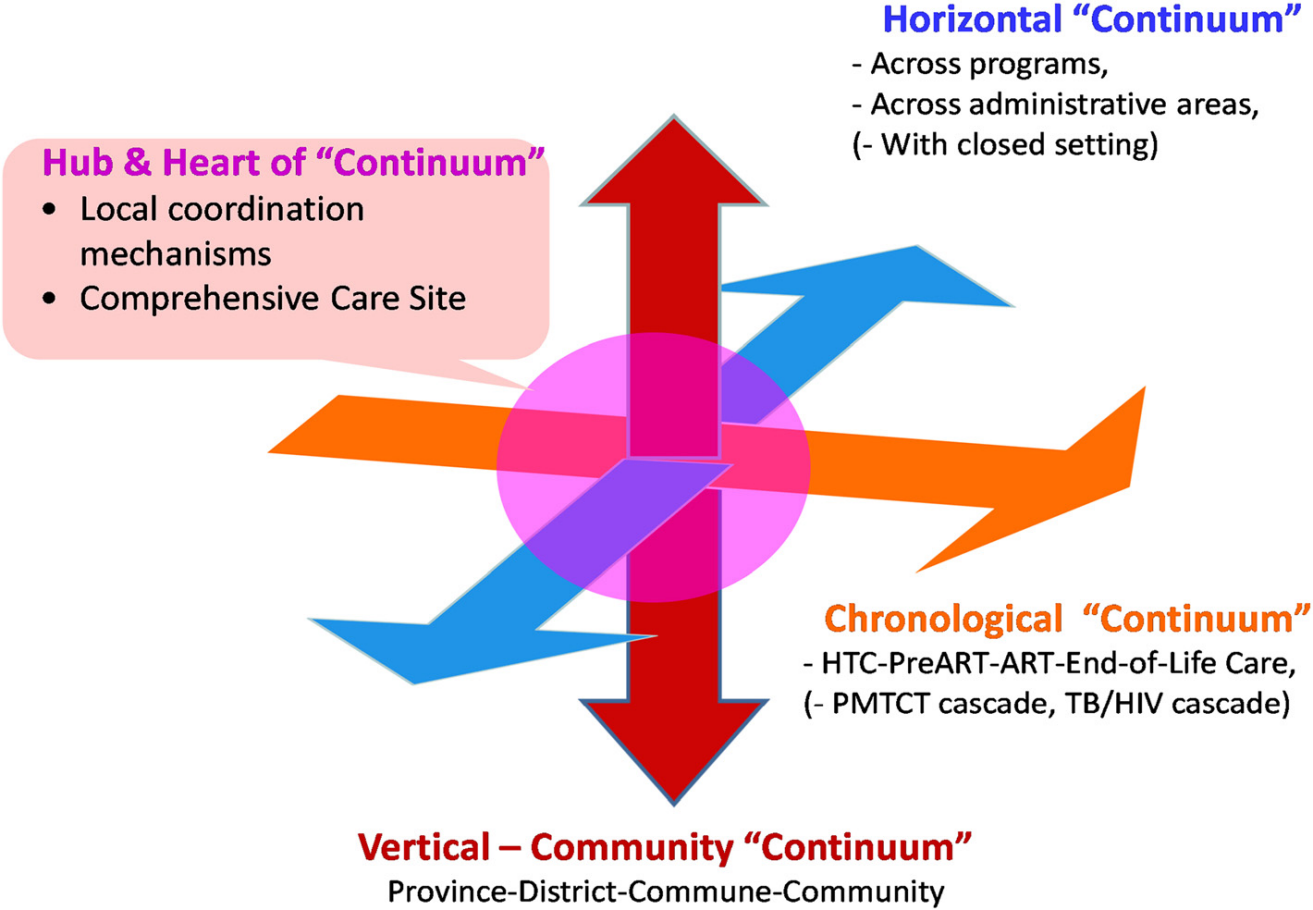
Analytical Framework of the Continuum of Prevention and Care (COPC).

We applied an analytical framework of the COPC [18] to assess HIV service delivery in the six countries in the Asia-Pacific region (Figure 1). The assessment looked into four continuum: 1) service linkages between key populations outreach and health facilities through HIV testing and counseling services (Vertical-Community Continuum); 2) chronic care provision including self-care, peer support and patient follow-up and tracking as well as recording systems throughout the stages of HIV diagnosis and HIV care and treatment (Chronological Continuum); 3) linkages and/or integration across HIV and other health services (Horizontal Continuum); and 4) comprehensive care sites involving PLHIV and CHBC (Hub and Heart of Continuum).

### Data collection

A regional team was formed to review national HIV health service delivery systems. The team consisted of officials from WHO Western Pacific Region, WHO South-East Asia Region, and FHI360 Asia Pacific Region as well as a consultant. The team adapted and adopted the COPC analytical framework that had been applied to a previous study in Vietnam [18].

Each country formed a review team which included national HIV program officials together with staff of WHO and/or FHI360 country offices. Assisted by an assigned focal point of the regional team, national HIV programs of respective country review teams identified and provided the information on the availability of HIV related services, approaches to link the services, and activities to monitor the performance. Data we collected were secondary program data from national HIV programs. Types of services covered included ART, HIV testing and counseling including voluntary counseling and testing (VCT) and provider-initiated testing and counseling (PITC) in TB and ANC services, CHBC, and HIV prevention for key populations. Global reports [31,32] were used to gather data on program performance including coverage of HIV testing, prevention and treatment as well as ART retention.

### Data analysis

For analyzing each continuum, data were examined to explore: contribution of service availability to the continuum; linking approaches taken to improve the continuum; and performance monitoring of the continuum.

Regarding the vertical-community continuum, we looked into: distribution and decentralization of VCT and HIV prevention for key populations (service availability); approaches to accelerate access to HIV testing and counseling and referral to care among key populations (linking approach); and HIV testing and counseling coverage among key populations and other related indicators (performance monitoring).

Concerning the chronological continuum, information examined include: distribution and decentralization of VCT and ART sites (service availability); approaches to improve retention from HIV testing to pre-ART enrolment, during pre-ART and during ART (linking approach); and status of monitoring systems on HIV testing to pre-ART, during pre-ART and during ART including national program data on ART retention and HIV drug resistance early warning indicators (performance monitoring).

On the horizontal continuum, data analysis focused on continuum between HIV, ANC and TB services for HIV testing and counseling. For this, we examined: distribution and decentralization of ANC services, TB services and HIV testing and counselling including VCT and PITC for pregnant women and TB cases (service availability); approaches to link between HIV, ANC and TB services for HIV testing and counseling (linking approach); and HIV testing coverage among pregnant women and TB cases, ARV coverage for PMTCT, and TB treatment and ART coverage among HIV positive TB patients (performance monitoring).

On the hub & heart of continuum, we analyzed the information on features and expansion status of comprehensive care sites including PLHIV involvement in ART sites and linkages with CHBC. Compiled data from each country was reviewed by the regional team, and additional information and clarifications were sought to complete the comparative analysis across the countries.

## Results

### Approaches to accelerate HIV diagnosis among key populations (Vertical-Community Continuum)

Service availability:

Availability of VCT was extensive covering all districts in all of the study countries except Vietnam and Myanmar (Table 2). In Vietnam, the number of districts with VCT was much fewer than the number of districts with outreach services for key populations. Quantifiable information on districts with outreach services for key populations was not available in Myanmar. In these countries, key populations living in districts without VCT had to travel to districts with VCT to access HIV testing and counselling services.

**Table 2:** **Access to HIV testing and counseling among key populations**

	**Cambodia**	**Myanmar**	**Nepal**	**PNG**	**Thailand**	**Vietnam**
Service availability						
Geographical areas of out-reach services*	46 districts for FSWs, 33 districts for MSM, 1 city for PWID	(Districts covered by NGOs for PWID and FSWs)	(For PWID, FSWs, MSM, TG and migrants)	National Capital District and Madang.	All districts	More than half of total districts for PWID and FSWs.
Total no. of VCT sites*	246	480	204	411	1316	272
Provinces with VCT sites*	24/24 provinces	14/14 provinces	14/14 zones	20/20 provinces	76/76 provinces	63/63 provinces
Districts with VCT sites*	77/77 districts	212/325 townships	75/75 districts	89/89 districts	878/878 districts	175/697 districts
VCT sites at lower than district (township) level*	166/997 health centers	Few	Few	121	Few	Few
Districts with VCT sites (%)*	100 (77/77 districts)	65 (212/325 townships)	100 (75/75 districts)	100 (89/89 districts)	100 (878/878 districts)	25 (175/697 districts)
Linking approach						
Approaches to accelerate access to testing and counseling and referrals to care among key populations*	−Peer educators refer key populations to VCT	−Peer educators refer key populations to VCT	−Peer educators refer key populations to VCT	−Peer educators refer key populations to VCT	−Peer educators refer key populations to VCT	−Peer educators refer key populations to VCT.
	−OST sites refer PWID to VCT	−OST sites refer PWID to VCT	−OST sites and drug treatment and rehabilitation centers refer PWID to VCT		−OST sites refer PWID to VCT	−OST sites refer PWID to VCT
	−On-site HIV testing initiated in hot spots for FSWs and MSM	−Drop-in centers refer key populations to VCT	−Drop-in centers refer key populations to VCT		−Drop-in centers refer key populations to VCT.	−Drop-in centers refer key populations to VCT
Performance monitoring						
HIV testing coverage among key populations (%)**						
FSW	81.5 (2010)	71.1 (2008)	54.6 (2011)	46.4 (2010)	50.4 (2010)	43.8 (2011)
MSM	34.0 (2010)	47.6 (2009)	42.0 (2009)	55.8 (2010)	29.2 (2010)	30.2 (2011)
PWID	35.3 (2007)	27.3 (2008)	21.4 (2011)	Not available	40.8 (2010)	29.1 (2011)

Source: *National HIV programs of 6 countries as of 2010, **AIDS Progress Report 2012 of Cambodia [19], Myanmar [20], Nepal [21], PNG [22], Thailand [23], Vietnam [24].

Remark: Province refers to the health administration, one level higher than so called district. District refers to the health administration level with the first referral-hospital (Operational district in Cambodia, Township in Myanmar, District in Nepal, PNG, Thailand, and Vietnam).

FSW: Female sex worker, MSM: Men having sex with men, PWID: People who inject drugs, TG: Transgender persons, OST: Opioid substitution therapy, VCT: Voluntary testing and counselling, CHBC: Community- and home-based care.

Linking approach:

In all countries, key populations were supposed to be referred to VCT from outreach services, opioid substitution therapy (OST) sites and drop-in centers. Innovative on-site HIV testing and counseling services were emerging to target key populations in the study countries. For example, in Cambodia, VCT staff started to visit drop-in centers in the hot spots to provide FSWs, MSM and TG with HIV testing and counseling as part of the ‘Community/Peer Initiated Testing and Counseling’ strategy. Other forms of point of care testing were being explored in other countries.

Performance monitoring:

All countries had HIV testing and counselling coverage data for FSW, MSM and PWID, except PNG, which had not reported injection drug use epidemic. The coverage among MSM and PWID appeared to be lower than that among FSW in five countries.

### Chronic care management (Chronological Continuum)

Services availability:

In Thailand, all districts had VCT and ART, in most cases within the same hospital compound (Table 3). In Vietnam, one-fourth of districts had both VCT and ART while the rest of the districts had neither VCT nor ART sites. In other countries, the number of districts/townships with VCT was far greater than that of districts/townships with ART sites. The proportion of districts/townships with VCT having ART sites was as low as 28% in Myanmar and 40% in Nepal. Except in Thailand, the proportion of districts/townships having ART sites was less than 70%; the proportion was as low as 18% in Myanmar, 24% in Vietnam and 40% in Nepal. These indicate that significant portions of PLHIV might need to travel across districts in order to enroll in pre-ART care and/or to retain in pre-ART/ART care in all countries but in Thailand. CHBC that supports adherence to ART and patient follow-up was available in all districts with ART in Cambodia while only 2 in 61 districts with ART in PNG.

**Table 3:** **Chronic care management**

	**Cambodia**	**Myanmar**	**Nepal**	**PNG**	**Thailand**	**Vietnam**
Service availability						
Total no. of ART sites	51	90	36	61	1014	217
Provinces with ART	21/24 provinces	13/14 provinces	13/14 zones	20/20 provinces	76/76 provinces	63/63 provinces
Districts with ART	44/77 districts	59/325 townships	30/75 districts	61/89 districts	878/878 districts	167/697 districts
Districts having ART (%)	57	18	40	69	100	24
Districts with VCT having ART (%)	57	28	40	69	100	95
District with CHBC /District with ART	44/44 (848/997 health center)	15 or more/59	13/30	2/61	400 or more/878	(185 teams)/167
Linking approach						
Testing and counseling (post-test counseling) to pre-ART care linkage	PLHIV referred to pre-ART care, but often in distant location. CHBC team offering referral support	PLHIV referred to pre-ART care, but often in distant location	PLHIV referred to pre-ART care, but often in distant location	PLHIV referred to pre-ART care, but often in distant location	PLHIV referred to pre-ART care. VCT and Pre-ART care located in the same facility	PLHIV referred to pre-ART care, but often in distant location
Generating a daily patient appointment list for ART	32 sites electronic, 19 sites paper-based system	Most sites paper-based system	Most sites paper-based system	4 sites electronic, 57 paper-based system	All sites electronic system	Most sites paper-based system
Performance monitoring						
Monitoring of pre-ART care through national program reporting	Enrolment: 7,391	Enrolment: 30,615	Enrolment: 15,443	Enrolment 2,541	Enrolment 28,264	Enrolment: 8,729
	Attrition: 2,249 (Lost and died monitored from 4^th^ quarter of 2010)	Attrition: not available	Attrition: not available	Attrition 414	Attrition 11,185	Attrition: Available only from selected sites
				−Lost 278	−Lost 6,892	
				−Died 136	−Died 4,293	
Monitoring of appointment keeping for ART	Annual facility survey in 42 sites, Local quality improvement in 16 sites.	Not yet operational	Not yet operational	Not yet operational	Annual facility survey in 38 sites	Annual facility survey in 30 sites
ART retention at 12-month (%)	93	87	N/A	78	83	82

Source: National HIV programs of 6 countries, AIDS Progress Report 2012 of Cambodia [19], Myanmar [20], Nepal [21], PNG [22], Thailand [23], Vietnam [24].

Remark: Province refers to the health administration, one level higher than so called district. District refers to the health administration level with the first referral-hospital (Operational district in Cambodia, Township in Myanmar, District in Nepal, PNG, Thailand, and Vietnam). CHBC: Community- and home-based care.

Linking approach:

In all countries, those diagnosed HIV positive were supposed to be referred from the sites offering post-test counselling to pre-ART care. In Cambodia, many of the newly diagnosed were enrolled in CHBC services that supported the patients to access pre-ART care. Supporting and promoting self-care of PLHIV to cope with HIV as chronic illness had been integrated into the training of health workers working at ART sites in all countries. The study countries had either electronic or paper-based systems to generate a list of patients who were supposed to attend the ART consultation on the appointment day.

Performance monitoring:

No monitoring system was established to assess how the process of referral from VCT to pre-ART enrolment was working in study countries. With regard to pre-ART care, Cambodia, PNG, Thailand, and Vietnam started monitoring of attrition from pre-ART care while this system was underdeveloped in other countries.

According to the national program reporting data, attrition of pre-ART patients were substantive particularly in Cambodia and Thailand. In Cambodia, several pre-ART care monitoring indicators were introduced as part of the country’s continuous quality improvement scheme.

As for patients already enrolled in ART, monitoring of appointment keeping was operational in Cambodia, Thailand and Vietnam. These countries introduced health facility survey methods to assess appointment keeping among other HIV drug resistance early warning indicators. Most ART sites surveyed in these countries achieved over 80% of appointment keeping. Data on ART retention at 12 months were available in all countries except Nepal. Cambodia, Myanmar, Vietnam and Thailand achieved over 80%.

### Linkages between HIV and TB or antenatal care (ANC) services (Horizontal Continuum)

**Figure 2:**
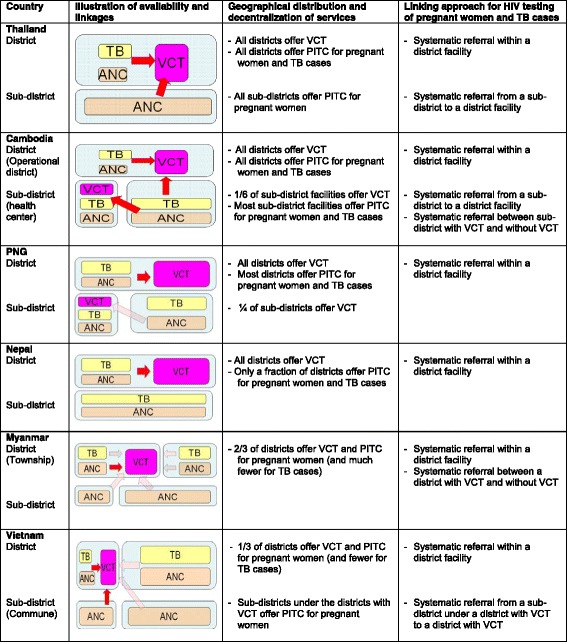
Operational linkages between HIV, tuberculosis, and maternal and child health services for HIV testing and counseling in 2010. Remark: Size of each box indicates the extent of availability (e.g. ¼ of sub-districts with VCT in PNG). A red arrow indicates systematic referral and a light-colored arrow indicates ad-hoc referral. ANC: Antenatal care. Source: National HIV Programs of 6 countries.

Services availability and linking approach:

Figure 2 illustrates geographical distribution and decentralization of ANC services, TB services, and HIV testing and counselling including VCT and PITC for pregnant women and TB cases, as well as their linkages across sub-district and district level.

In Thailand, VCT, TB, and ANC services were available and linked within the same district hospitals. In addition, Thailand established the system to send blood samples from all sub-district health centers to district hospitals in support of PITC for pregnant women. Cambodia established the system for sub-district level health centers, including those that did not offer VCT, to send blood samples taken from both TB patients and pregnant women to health facilities with VCT.

In Nepal, PNG, and Myanmar, VCT, TB and ANC services were located in the same district/township level facilities or there were linkages between these services across districts. However, most sub-district level TB and ANC services were not systematically linked to HIV testing and counselling services. In Vietnam, one-fourth of districts had linkages between TB and ANC services and HIV testing and counselling services. That is, these linkages existed only in the districts that offered VCT. PITC for pregnant women at sub-district (commune) level was introduced under the districts with VCT only. In these districts, district level staff visited sub-district level facilities to provide HIV testing and counselling when ANC services were operating.

Performance monitoring:

All countries had coverage data on HIV testing for pregnant women, HIV testing for TB cases, ARV for pregnant women to prevent mother-to-child transmission, and ART and TB treatment for HIV positive TB cases (Table 4). HIV testing and counseling coverage among pregnant women and TB cases were more than 70% in Thailand and Cambodia while around 50% or less in other countries. ARV coverage for PMTCT was highest (94%) in Thailand, followed by Cambodia, Myanmar and Vietnam (44-64%). It was as low as 12% in Nepal and PNG. TB treatment and ART coverage among HIV positive TB patients were around 30% in Cambodia, Vietnam, Thailand and PNG, and no data were available in Myanmar and Nepal.

**Table 4:** **Performance monitoring of linkages between HIV, TB and maternal, newborn and child health (MNCH)**

	**Cambodia**	**Myanmar**	**Nepal**	**PNG**	**Thailand**	**Vietnam**
Pregnant women who know HIV status (%), 2010*	74	35	13	24	94	52
TB patients who know HIV status (%), 2010**	77	3	NA	7	77	43
ARV coverage for pregnant women to prevent mother-to-child transmission (%), 2011***	64	55	12	12	94	44
HIV positive TB patients who received ART and TB treatment (%), 2011***	33	NA	NA	25	26	30

Source: *Global HIV/AIDS response: epidemic update and health sector progress towards universal access: progress report 2011 [31].

**Global tuberculosis control, WHO Report 2011 [32].

***National HIV programs of 6 countries, AIDS Progress Report 2012 of Cambodia [19], Myanmar [20], Nepal [21], PNG [22], Thailand [23], Vietnam [24].

### ART sites offering comprehensive care through the involvement of PLHIV and CHBC teams (Hub and Heart of Continuum)

All the countries had national guidance on CHBC and/or PLHIV involvement (Table 5). However, the involvement of PLHIV as care providers and peer supporters was more systematic in some countries than others. In Cambodia, for example, most ART sites established ‘MMM’ (Center for Friends Help Friends) that were managed by PLHIV. The ‘MMM’ members organized monthly meetings involving PLHIV, CHBC teams and local stakeholders.

**Table 5:** **ART sites providing comprehensive care through the involvement of PLHIV and links to CHBC**

	**Cambodia**	**Myanmar**	**Nepal**	**PNG**	**Thailand**	**Vietnam**
Linking approach						
National guidance and framework	−Framework and SOPs on continuum of care	Strategy on comprehensive continuum of care for PLHIV	−Strategy to initiate CHBC	−Strategy of greater involvement of PLHIV	−Policy of National Security Office to support PLHIV network	−Action plan on care and treatment
	−SOPs for ‘MMM’ (center for friends help friends) and CHBC		−Guidelines and SOPs for CHBC			−ART protocol
						−SOPs for CHBC
Features of collaboration between ART sites, PLHIV and CHBC	−‘MMM’ established inside ART sites and managed by a few PLHIV	−NGO clinics covering about 75% of PLHIV on ART	−PLHIV workers of NGOs visit ART sites on clinic days to complement services provided by health workers	−ART sites established continuum of care centers as pilot project	−Comprehensive Continuum of Care (CCC) Centers run by PLHIV	−ART sites involving PLHIV as member of ART team and facilitating PLHIV peer support groups
	−Monthly meetings of ‘MMM’ involving patients, health workers and often hospital management	−In all NGO clinics, PLHIV working as part of care team	−Most CHBC teams involve PLHIV and are based at NGO-run HIV prevention and care centers	−PLHIV peer educators in ART sites	−CCC Centers located inside ART sites in some areas, outside in other areas	−A number of CHBC models including ART sites based; Stand-alone model run by PLHIV groups and local NGOs; Led by Women’s Union; and Commune health station based.
	−PLHIV working as part of ART team	−PLHIV having some role in government run clinics too		−The continuum of care centers linked to CHBC teams	−CHBC led by PLHIV as part of activities of CCC Centers	
	−NGO-led CHBC teams involving PLHIV and attending ‘MMM’ meetings and local coordination meetings	−Local coordination meetings involving CHBC				
Scale	−Most ART sites having MMM	−PLHIV as care provider in all NGO run clinics	−All ART sites supported by PLHIV workers	−4 ART sites established the centers	−367 CCC Centers operational	−More than 100 ART sites involve PLHIV as member of ART teams and linked to CHBC
	−356 CHBC teams linked to 848 sub-district health centers	−CHBC at least in 15/325 townships	−More than half of ART sites linked to CHBC	−5 ART sites having PLHIV peer educators	−CHBC at least in 400/878 districts	−185 CHBC teams
			−CHBC in 13/75 districts	−CHBC in 2/89 districts		

Remark: SOPs: Standard operating procedures, CHBC: Community- and home-based care.

Source: National HIV programs of 6 countries as of 2010.

In Thailand, PLHIV were playing a vital role in providing HIV care and treatment at least in 367 government hospitals in 878 districts in collaboration with the Comprehensive Continuum of Care Centers led by PLHIV. CHBC services in Nepal were linked with NGO-managed HIV prevention and care services, as well as with provincial/zonal hospitals in areas where HIV was most prevalent. In PNG, PLHIV involvement in CHBC was promoted only in a few pilot sites.

## Discussion

Our analysis on the four continuum revealed that HIV service availability and linking approaches served as supporting factors in some cases while constrained in others.

Regarding the vertical-community continuum, HIV testing and counseling coverage among key populations ranged from 20% to 80% across countries and across populations. In five countries, HIV testing and counseling coverage were low among MSM and PWID compared with that among FSW. Low coverage in Myanmar and Vietnam could be partly due to geographical distance from hot spots to VCT located in other districts. To effectively serve key populations, it is critical to make a range of relevant HIV services convenient to their communities [33,34]. However, this becomes challenging when donor funded projects for prevention among key populations and those for diagnosis and treatment have different geographical scope. In Myanmar and Vietnam, for example, limited access to HIV testing and counseling by key populations may be related to geographical discrepancy in service provision and possible sub-optimal linkages across these projects [18,35-37]. Among different key populations, countries have more extensive experiences in providing services for FSW than for MSM and PWID in Asia and the Pacific [33,38]. HIV testing coverage among FSW tends to be higher than those among MSM and PWID in these countries [1].

The chronological continuum showed a visible progress. All of the countries established case management procedures for those on ART in line with the chronic care principles [39]. They then achieved a high level of ART retention. However, our study revealed significant attrition from and lack of data on the process from HIV diagnosis through pre-ART care. It also found low proportion of districts with VCT having pre-ART care and/or low proportion of districts having pre-ART care in all countries except Thailand. In these countries, no information was available to know how effectively referral services were working from HIV testing to pre-ART care. Monitoring of retention in pre-ART care was not well established in all study countries. In the last decade, global efforts to expand HIV care and treatment have prioritized ART provision while less attention has been paid to the process between HIV testing and ART initiation [40-43]. Many studies reported significant attrition from the process [44-46]. According to a systematic review, barriers to accessing pre-ART care include transport costs and distance, fear of disclosure, long waiting times, and shortage of health workers [47]. Activities that may decrease attrition include streamlining services to minimize facility visits, introducing point-of-care CD4 testing and peer support, and providing incentives [48]. In our study countries, little was known about access to and retention in pre-ART care. A possible challenge was the requirement for many PLHIV to travel long distance to access pre-ART care in other districts.

Our analysis on the horizontal continuum identified a number of challenges. HIV testing and counselling coverage among pregnant women and TB cases was less than 70% except in Thailand and Cambodia. ARV coverage for PMTCT was less than 70% in all countries except Thailand. TB treatment and ART coverage among HIV positive TB patients was low at around 30% or data were unavailable in all of these countries. In Thailand, all districts had VCT, TB and ANC services and established systematic linkages with sub-district health centers for PITC. In Cambodia, limited availability of VCT was complemented by extensive blood sample referral systems between health centers and district hospitals and across districts. In Myanmar, Nepal, PNG and Vietnam, PITC for TB cases and pregnant women was constrained by sub-optimal linkages between district and sub-district levels and/or between districts with VCT and districts without VCT. A challenge for PMTCT and TB/HIV collaborative activities is expanding PITC based on local context and available resources [41]. Sub-optimal linkages between districts with and without VCT in Vietnam appeared to be related to operations of donor-funded projects [18]. Where donor funded projects exited, VCT and PITC were available at the district and sub-district levels. However, districts not supported by these projects had no VCT/PITC or linkages with districts supported by the projects. To address the issue of linkages between district and sub-district levels, national HIV programs of Thailand and Cambodia proactively guided local health facilities across the country to establish blood sample referral systems between district and sub-district levels [49,50].

The progress on the hub and heart of the continuum varied across the study countries. Comprehensive care sites had been expanded more systematically in Cambodia and Thailand than in other countries. All the studied countries introduced certain mechanisms to involve PLHIV in providing care and treatment services and link them with CHBC, but to varying degrees. PLHIV and CHBC networks were established during the 1990’s prior to the ART introduction in Thailand and Cambodia [15]. These countries proactively used existing PLHIV and CHBC networks to expand ART services [49,50]. In other countries, these networks were developed and expanded along with the ART scale-up [15,18,51,52].

Our study revealed some similarities and considerable variations in HIV service availability, linking approaches and performance monitoring across the study countries. The COPC analytical framework can be a useful tool for respective national HIV programs to critically review the current status of HIV service delivery. The framework can also help countries identify evidence-informed measures when adapting global guidance and recommendations on decentralizing, linking and integrating HIV services [53,54].

As observed in our study, the COPC analytical framework reflects innovative features of HIV health service delivery. These features could benefit the rest of heath care system including the non-communicable disease services [55,56]. For example, the national HIV programs established the following chronic disease management systems [57,58]. Services were integrated within public health care facilities and liked to the communities. Patients play a central role in promoting self-care, treatment adherence, and peer support by reaching affected communities and involving them as co-service providers. Longitudinal patient follow-up systems have been introduced with registers and individual patients’ cards and files [7]. Furthermore, linkages with other relevant services have been developed to meet the multiple needs of patients. The COPC analytical framework could be used to engage a wide range of stakeholders in the health sector to adapt and adopt these HIV service delivery features in order to strengthen the overall health care system and expand non-communicable disease services. This process may in turn promote HIV service sustainability by integrating the HIV chronic care management into the overall health care system [3].

The main limitation of this study was that the reviewed literature included unpublished information obtained through the National HIV Program Office of each country. It was therefore not easy to ascertain the same level of data quality across the study countries. Also, interpretation of the findings might not be completely objective as several co-authors have been involved in policy and program development for expanding service delivery in the study countries. However, by assigning a focal point of the regional team to work with the co-authors in each country, we tried to examine data critically and reflect each issue presented in this paper from multi-country perspectives. It should also be noted that active participation of co-authors in this study increased the likelihood of utilizing the findings to strengthen health sector response to HIV. Study countries were not chosen randomly as we intended to compare service delivery models among the countries which were experiencing concentrated HIV epidemics and willing to participate in the joint exercise to improve HIV case detection and retention in care. Nevertheless, our results suggest that the COPC framework might be useful beyond the study countries.

## Conclusions

This study identified similarities and variations in service availability and linking approaches across the countries. Similarities include well established case management procedures for those on ART and underdeveloped process between HIV testing and ART initiation. Considerable variations were found in availability of VCT and pre-ART/ART care at district level; linkages between district and sub-district levels and between districts with VCT and districts without VCT; and extent of involving PLHIV in providing care and treatment services and linking them with CHBC. HIV service availability patterns and the linking approaches served as supporting factors in some cases while as constraints in others for the performance of each continuum. Furthermore, the continuum appeared to be more coherent in some countries than in others.

Based on our findings, the study countries could consider the following options. For the vertical-community continuum, it would be useful to review linkages between prevention and HIV testing services. Special attention need be paid to service linkages across multiple projects. Outreach HIV testing should be introduced in line with global recommendations [53,59]. To improve the chronological continuum, it is critical to enhance the monitoring of patient flow from HIV testing to ART initiation. Study countries should explore ways to minimize attrition from this process learning from other countries [49,60,61]. Regarding the horizontal continuum, measures need to be taken to strengthen linkages between districts and between district and sub-district levels according to local need [59,62-64]. PLHIV and CHBC networks need to be better engaged and supported as part of the hub and heart of continuum, and in enabling retention in HIV care services. They could also help address emerging needs such as ART as prevention for discordant couples [65,66].

As a result of this study, opportunities now exist for cross-fertilization among these six countries as well as national HIV program reviews to adjust geographical distribution and decentralization of HIV services and to systematically strengthen multiple linkages. These efforts will promote early HIV diagnosis, early access to and retention in pre-ART care and long-term retention on ART. Our study results suggest that the COPC analytical framework could help each country identify evidence-informed measures when applying global recommendations on decentralizing, integrating and linking HIV services to move towards ‘Zero AIDS-related deaths’.

## Abbreviations

ANC: Antenatal care
ART: Antiretroviral treatment
CHBC: Community- and home-based care
COPC: Continuum of prevention and care
MSM: Men who have sex with men
MARPs: Most-at-risk populations
MNCH: Maternal, newborn and child health
NGOs: Non-governmental organizations
OST: Opioid substitution therapy
PITC: Provider-initiated testing and counseling
PWID: People who inject drugs
PLHIV: People living with HIV
FSWs: Female sex workers
TB: Tuberculosis
TG: Transgender
VCT: Voluntary counseling and testing

## References

[1] Joint United Nations Programme on HIV/AIDS (2013). HIV in Asia and the Pacific: UNAIDS Report 2013.

[2] Joint United Nations Programme on HIV/AIDS (2010). Getting to Zero: UNAIDS Strategy 2011–2015.

[3] World Health Organization (2011). Global Health Sector Strategy on HIV/AIDS 2011–2015.

[4] The Global Fund to Fight AIDS, Tuberculosis and Malaria (2011). The Global Fund Annual Report 2011.

[5] World Health Organization and Joint United Nations Programme on HIV/AIDS (2011). The treatment 2.0 framework for action: catalysing the next phase of treatment, care and support.

[6] Hirnschall G, Schwartländer B (2001). Treatment 2.0: catalysing the next phase of scale-up. Lancet.

[7] Srikantiah P, Ghidinelli M, Bachani D, Chasombat S, Daoni E, Mustikawati DE (2010). Scale-up of national antiretroviral therapy programs: progress and challenges in the Asia Pacific region. AIDS.

[8] Legido-Quigley H, Montgomery CM, Khan P, Fakoya A, Getahum H, Grant AD. Integrating tuberculosis and HIV services in Low- and middle-income countries: a systematic review. Geneva: World Health Organization; 2010. Available at: http://r4d.dfid.gov.uk/pdf/outputs/hiv_aids/legidoquigley_etal_integrtuberculosis.pdf.. Accessed 28 November 2011. 10.1111/tmi.12029 23217030

[9] World Health Organization, United Nations Children’s Fund, United Nations Population Fund and Joint United Nations Programme on HIV/AIDS (2008). Asia-pacific operational framework for linking HIV/STI services with reproductive, adolescent, maternal, and newborn and child health services.

[10] World Health Organization, United States Agency for International Development and Family Health International (2009). Strategic considerations for strengthening the linkages between family planning and HIV/AIDS policies, programs, and services.

[11] Narain JP, Chela C, van Praag E (1998). Planning and implementing HIV/AIDS care programmes: a step-by-step approach.

[12] National Center for HIV/AIDS, Dermatology and STD (NCHADS), Ministry of Health Cambodia (2003). The continuum of care for people living with HIV/AIDS: operational framework in Cambodia.

[13] World Health Organization Regional Office for the Western Pacific (2004). HIV/AIDS care and treatment: guide for implementation.

[14] World Health Organization Regional Office for the Western Pacific and National Center for HIV/AIDS, Dermatology and STD (NCHADS) (2006). The continuum of care for people living with HIV/AIDS in Cambodia: linkages and strengthening in the public health system. Case study.

[15] Green K, McPherson R, Fujita M, Lo YR, Natpratan C, van Praag E (2007). Make it happen at the local level: Establishing the continuum of care. Scaling up the continuum of care for people living with HIV in Asia and the Pacific: a toolkit for implementers.

[16] Gardner EM, McLees MP, Steiner JF, del Rio C, Burman WJ (2011). The spectrum of engagement of HIV care and its relevance to test-and-treat strategies for prevention of HIV infections. Clin Infect Dis.

[17] World Health Organization Regional Offices for the Western Pacific and South-East Asia (2014). Metrics for monitoring the cascade of HIV testing, care and treatment services in Asia and the pacific.

[18] Fujita M, Poudel KC, Do TN, Bui DD, Nguyen VK, Green K (2012). A new analytical framework of ‘continuum of prevention and care’ to maximize HIV case detection and retention in care in Vietnam. BMC Health Serv Res.

[19] National AIDS Authority, Cambodia (2012). Cambodia Country Progress Report: Monitoring the Progress Towards the Implementation of the Declaration of Commitment on HIV and AIDS; Reporting Period January 2010 – December 2011.

[20] National AIDS Program, Myanmar (2012). Global AIDS Response Progress Report Myanmar: Reporting period January 2010 – December 2011.

[21] National Center for AIDS and STD Control, Ministry of Health and Population Nepal (2012). Country Progress Report 2012.

[22] National AIDS Council Secretariat (2012). Global AIDS report 2012: country progress report Papua New Guinea: reporting period January 2010 – December 2011.

[23] National AIDS Prevention and Alleviation Committee (2012). Thailand AIDS Response Progress Report 2012: Reporting period 2010 – 2011.

[24] National Committee for AIDS, Drugs and Prostitution Prevention and Control (2012). Viet Nam AIDS Response Progress Report 2012: Following up the Implementation to the 2011 Political Declaration on HIV/AIDS: Reporting Period January 2010 – December 2011.

[25] Liebowitz B, Brody EM (1970). Integration of research and practice in creating a continuum of care for the elderly. Gerontology.

[26] Kerber KJ, Graft-Johnson JE, Bhutta ZA, Okong P, Starrs A, Lawn JE (2007). Continuum of care for maternal, newborn, and child health: from slogan to service delivery. Lancet.

[27] Anderson S (1994). Community responses to AIDS. World Health Forum.

[28] Jackson H, Kerkhoven R (1995). Developing AIDS care in Zimbabwe: a case for residential community centers?. AIDS Care.

[29] Schietinger H, Sanei L (1998). A Continuum of HIV/AIDS Prevention and Care. Systems for Delivering HIV/AIDS Care and Support, Discussion Paper on HIV/AIDS Care and Support No.8.

[30] International HIV/AIDS Alliance (2000). Linking prevention and care. Care, Involvement and Action: Mobilising and Supporting Community Responses to HIV/AIDS Care and Support in Developing Countries.

[31] World Health Organization, Joint United Nations Programme on HIV/AIDS, United Nations Children’s Fund (2011). Global HIV/AIDS Response: Epidemic Update and Health Sector Progress Towards Universal Access: Progress Report 2011.

[32] World Health Organization (2011). Global Tuberculosis Control: WHO Report 2011.

[33] Commission on AIDS in Asia (2008). Redefining AIDS in Asia: Crafting an Effective Response.

[34] World Health Organization (2014). Key factors to consider when providing services for All Key populations. Consolidated guidelines on HIV prevention, diagnosis, treatment and care for Key populations.

[35] Zhang L, Maher L, Quang DP, Higgs P, Ngo DA, Bui HD (2013). Evaluation of a decade DFID and World Bank supported HIV and AIDS programmes in Vietnam from 2003 to 2012.

[36] Global HIV/AIDS Initiatives Network. Briefing sheet 3: The challenge of coordination. Global HIV/AIDS Initiatives Network; 2008. [http://www.who.int/alliance-hpsr/researchsynthesis/AllianceHPSR_GHIN_ChallengeCoordination_BS3.pdf]

[37] National AIDS Programme, Ministry of Health (2012). Progress Report 2012: National Strategic Plan for HIV/AIDS in Myanmar.

[38] McMillan K (2013). Sex work and HIV/STI prevention in the pacific region, including analysis of the needs of, and lessons learnt from, programs in four selected countries.

[39] Bodenheimer T, Wagner EH, Grumbach K (2002). Improving primary care for patients with chronic illness: the chronic care model, Part 2. JAMA.

[40] World Health Organization and Joint United Nations Programme on HIV/AIDS (2006). Progress on global access to HIV antiretroviral therapy: a report on 3 by 5 and beyond, March 2006.

[41] World Health Organization, Joint United Nations Programme on HIV/AIDS and UNICEF (2009). Towards Universal Access: Scaling up Priority HIV/AIDS Interventions in the Health Sector: Progress Report, September 2009.

[42] World Health Organization, Joint United Nations Programme on HIV/AIDS and UNICEF (2011). Global HIV/AIDS Response: Epidemic Update and Health Sector Progress Towards Universal Access: Progress Report 2011.

[43] World Health Organization (2013). Global update on HIV treatment 2013: results, impact and opportunities: WHO report in partnership with UNICEF and UNAIDS.

[44] Rosen S, Fox MP (2011). Retention in HIV care between testing and treatment in Sub-Saharan Africa: a systematic review. PLoS Med.

[45] Mugglin C, Estill J, Wandeler G, Bender N, Egger M, Gsponer T (2012). Loss to programme between HIV diagnosis and initiation of antiretroviral therapy in sub-Saharan Africa: systematic review and meta-analysis. Trop Med Int Health.

[46] Kranzer K, Govindasamy D, Ford N, Johnston V, Lawn SD (2012). Quantifying and addressing losses along the continuum of care for people living with HIV infection in sub-Saharan Africa: a systematic review. J Int AIDS Soc.

[47] Govindasamy D, Ford N, Kranzer K (2012). Risk factors, barriers and facilitators for linkages to antiretroviral therapy care: a systematic review. AIDS.

[48] Govindasamy D, Meghij J, Negussi EK, Baggaley RC, Ford N, Kranzer K (2014). Interventions to improve or facilitate linkage to or retention in pre-ART (HIV) care and initiation of ART in low- and middle- income settings: a systematic review. J Int AIDS Soc.

[49] Mean CV, Fujita M, Tung R, Mao TE, Seng S, Samreth S (2014). Achieving universal access and moving towards elimination of new HIV infections in Cambodia. J Int AIDS Soc.

[50] Ministry of Public Health, Thailand and World Health Organization Regional Office for South-East Asia (2005). External review of the health sector response to HIV/AIDS in Thailand.

[51] World Health Organization Regional Office for South-East Asia (2006). Review of the Myanmar National AIDS Programme 2006.

[52] Papua New Guinea National AIDS Council Secretariat and Partners (2008). UNGASS 2008 Country Progress Report: Papua New Guinea. Reporting Period: January 2006–December 2007.

[53] World Health Organization (2013). Consolidated guidelines on the Use of antiretroviral drugs for treating and preventing HIV infection: recommendations for a public health approach.

[54] World Health Organization (2014). Consolidated guidelines on HIV prevention, diagnosis, treatment and care for Key populations.

[55] Rabkin M, Kruk ME, El-Sadr WM (2012). HIV, aging and continuity care: strengthening health systems to support services for noncommunicable diseases in low-income countries. AIDS.

[56] Rabkin M, El-Sadr WM (2011). Why reinvent the wheel? Leveraging the lessons of HIV scale-up to confront non-communicable diseases. Glob Public Health.

[57] Poudel KC, Fujita M, Green K, Poudel-Tandukar K, Jimba M (2011). Non-communicable diseases in Southeast Asia. Lancet.

[58] Green K, McPherson R, Fujita M, Lo YR, Natpratan C, van Praag E (2007). Continuum of care country profiles. Scaling up the continuum of care for people living with HIV in Asia and the Pacific: a toolkit for implementers.

[59] World Health Organization (2012). Service delivery approaches to HIV testing and counselling (HTC): a strategic HTC policy framework.

[60] Jani IV, Sitoe NE, Alfai ER, Chongo PL, Quevedo JI, Rocha BM (2011). Effect of point-of-care CD4 cell count tests on retention of patients and rates of antiretroviral therapy initiation in primary health clinics: an observational cohort study. Lancet.

[61] Mtapuri-Zinyowera S, Chideme M, Mangwanya D, Mugurungi O, Gudukeya S, Hatzold K (2010). Evaluation of the PIMA Point-of-Care CD4 Analyzer in VCT Clinics in Zimbabwe. J Acquir Immune Defic Syndr.

[62] Hensen B, Baggaley R, Wong VJ, Grabbe KL, Shaffer N, Lo YR (2011). Universal voluntary HIV testing in antenatal care settings: a review of the contribution of provider-initiated testing and counselling. Trop Med Int Health.

[63] Matida LH, Santos NJ, Ramos AN, Gianna MC, de Silva MH, Domingues CS (2011). Eliminating vertical transmission of HIV in São Paulo, Brazil: progress and challenges. J Acquir Immune Defic Syndr.

[64] World Health Organization, Joint United Nations Programme on HIV/AIDS (2007). Guidance on provider initiated HIV testing and counselling in health facilities.

[65] Cohen MS, Chen YQ, McCauley M, Gamble T, Hosseinipour MC, Kumarasamy N (2011). Prevention of HIV-1 infection with early antiretroviral therapy. N Engl J Med.

[66] World Health Organization (2012). Guidance on couples HIV testing and counseling including antiretroviral therapy for treatment and prevention in serodiscordant couples: recommendations for a public health approach.

